# Cerebrospinal Fluid Leak Prevention in Intradural Spine Surgery: A Long Series Analysis of Closure with Non-Penetrating Titanium Clips

**DOI:** 10.3390/brainsci14121223

**Published:** 2024-12-03

**Authors:** Leonardo Anselmi, Carla Daniela Anania, Maria Cleofe Ubezio, Generoso Farinaro, Donato Creatura, Alessandro Ortolina, Massimo Tomei, Ali Baram, Maurizio Fornari

**Affiliations:** 1 Department of Neurosurgery, IRCCS Humanitas Research Hospital, Via Manzoni 56, 20089 Rozzano, Italy; leonardo.anselmi@humanitas.it (L.A.); generoso.farinaro@humanitas.it (G.F.); donato.creatura@humanitas.it (D.C.); alessandro.ortolina@humanitas.it (A.O.); massimo.tomei@humanitas.it (M.T.); ali.baram@humanitas.it (A.B.); maurizio.fornari@humanitas.it (M.F.); 2 Department of Biomedical Sciences, Humanitas University, Via R.L Montalcini 2, 20072 Pieve Emanuele, Italy; mariacleofe.ubezio@st.hunimed.eu

**Keywords:** AnastoClip, non-penetrating titanium clip, durotomy, dural closure, CSF, dural tears, spinal intradural tumors surgery, spine surgery

## Abstract

**Background/Objectives**: Postoperative cerebrospinal fluid (CSF) fistulas remain a significant concern in spinal neurosurgery, particularly following dural closure. The incidence of dural tears during spinal surgery is estimated between 1.6% and 10%. While direct suturing remains the gold standard, it has a failure rate of 5–10%. Various materials and techniques have been used to enhance dural closure. This study aims to assess the effectiveness of non-penetrating titanium clips (AnastoClip^®^) for dural closure in intradural spinal lesion surgeries. **Methods**: A prospective analysis was conducted on 272 patients who were operated on for intradural spinal lesions from August 2017 to December 2023. Dural closure was performed using non-penetrating titanium clips with sealant, and, in select cases, autologous grafts. Postoperative care included early mobilization and routine MRI to assess outcomes. A comparative analysis was performed with a cohort of 81 patients treated with traditional sutures. **Results**: Among the 272 patients, postoperative CSF leaks occurred in 32 cases (11.76%), requiring various management approaches. Thirteen cases required surgical revision, while others resolved with external lumbar drainage or fluid aspiration. Compared to the suture group, which had a fistula rate of 23.46%, the titanium clip group had a significantly lower fistula rate. Logistic regression analysis did not find statistically significant associations between fistula risk and clinical factors. **Conclusions**: Non-penetrating titanium clips provide an effective alternative to sutures for dural closure, reducing CSF leak rates. They preserve dural integrity, reduce operative time, and avoid imaging artifacts, making them a viable advancement in spinal surgery with outcomes comparable to, or better than, traditional techniques.

## 1. Introduction

Avoiding postoperative cerebrospinal fluid fistulas has been a challenge in neurosurgery for a long time [1]. Dural opening in spinal surgery may be intentional, as in intradural procedures, or incidental, as in dural tears, with dural opening occurring in 1.6% to 10% of cases in other types of spinal surgery [2]. Over time, various methods have been proposed to obtain a watertight closure including different suture techniques (interrupted or continuous) [3] and additional sealing materials and grafts such as autologous fat or muscle [4,5]. Even today, there is no consensus on the most appropriate closure method or postoperative management approach to minimize the risk of dural opening. Nowadays, the primary accepted goal worldwide is to obtain the direct suture whenever possible in order to reduce the cerebrospinal fluid leakage with minimal risk; however, even with additional sealants, the use of direct sutures is still associated with a 5% to 10% failure rate [6].

Over the years, a lot of innovative and alternative methods have been described with the aim of obtaining a watertight closure that could be performed in a safer, easier, and more rapid way. Based on the first studies in 2010 and the more recent paper of Kiyoshi Ito in 2017, we have introduced the use of non-penetrating titanium clips (the AnastoClip^®^ AC Closure System.LeMaitre Vascular S.R.L. Via Clemente Prudenzio 14/16 20138 Milano (MI), Italy) at our department for dural closure in both intentional and incidental durotomy [7]. We collected a series of almost 300 surgical procedures for intradural spinal lesions operated on with this closure technique. We also gathered a prospective collection of all cases to analyze the feasibility and the effectiveness of this novel technique in terms of the prevention of dural fistulas and the reduction of the reintervention rate. To achieve this result, we made a comparison with a retrospective analysis of a series of patients operated on from 2015 to 2017.

In this paper, we present the surgical technique and the results of both the prospective collection and the comparative analysis.

## 2. Materials and Methods

**Figure 1:**
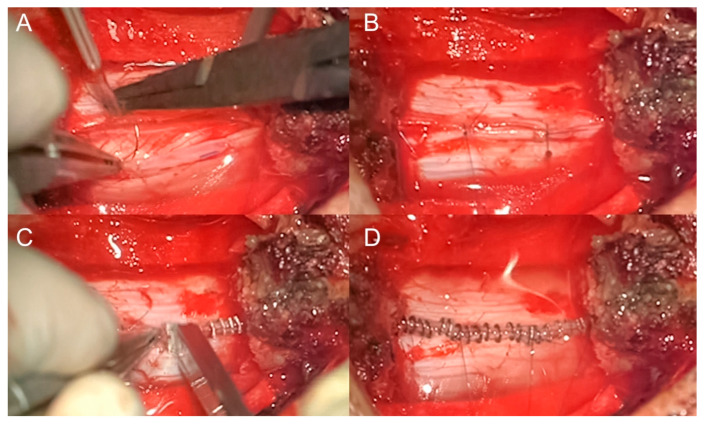
Closure technique for intentional durotomy. For the closure of the dura mater, we use two to three stitches with Prolene 6-0 suture thread and a curved needle: two stitches are placed at the edges of the dural opening, with one or two additional stitches in the middle, depending on the size of the opening (**A**,**B**). Titanium clips are then applied to secure the dural closure, followed by the application of a sealant (**C**,**D**).

We prospectively analyzed a series of patients operated on at our Neurosurgery Department from August 2017 to December 2023 for resection of intradural spinal lesions both intra- and extramedullary. The study includes adult patients who underwent resection of intradural spinal tumors with an intact dura mater, where dural closure was achieved using non-penetrating titanium clips. Excluded from the study were patients with a history of prior surgery for intradural spinal lesions, those who experienced incidental durotomy requiring suturing, and patients whose postoperative dural defects necessitated the use of artificial dural patches. All patients underwent the same closure technique and postoperative cares protocol. In detail, dural closure was achieved by the placement of non-penetrating titanium clips (AnastoClip^®^) and sealant; patches (gelatin sponge or Tachoseal^®^) or autologous graft (adipose or muscular tissue) were applied in selected cases. Patients deemed at risk were kept in bed for 24 to 48 h, while the majority were mobilized the day after surgery. The surgical technique and postoperative cares protocol are shown in Figure 1 and detailed in Appendix A.

Clinical data such as age, sex, BMI, operative data including the size of dural opening (dimensionally equivalent to the number of vertebrae), vertebral segment (divided in cervical, dorsal, lumbar, and sacral), and pathological data such as the histological type of tumor were analyzed. Additionally, the number of fistulas and the method of resolution were evaluated, including reintervention, placement of an external lumbar drainage (ELD) catheter, reintervention plus ELD, or spontaneous resolution after aspiration of the collection and compressive medication. For a comparative analysis, a similar population of patients undergoing dural closure with standard sutures operated on from January 2015 to December 2017, prior to the introduction of clips, was retrospectively analyzed.

To reduce biases, we did not analyze patients with incidental durotomy repaired with titanium clips.

The study was approved by our institutional ethics committee (N° 31/24).

## 3. Results

We conducted a comprehensive analysis involving 272 patients, encompassing 120 males and 152 females, with a mean age of 56 years (range: 11–89). Within this cohort, 234 individuals presented with extramedullary lesions, 33 with intramedullary lesions, and 5 with combined intra- and extramedullary involvement. Anatomical distribution revealed 57 cervical, 105 thoracic, 108 lumbar, and 2 sacral lesions. Dural exposure ranged from one level in 69 cases, two levels in 160 cases, three levels in 30 cases, to exceeding three levels in 13 cases. Among these patients, 32 cases (11.76%) manifested postoperative cerebrospinal fluid (CSF) leaks (13 cases with loss of fluid from the wound and 19 cases with subcutaneous collection), necessitating careful management. Surgical revision was required in 13 instances, while resolution was achieved in 5 cases through external lumbar drainage alone, in 4 cases with a combination of surgery and subsequent external lumbar drainage, and in 10 cases through fluid aspiration coupled with compressive dressing. Notably, one patient required a blood patch due to the intricacies of dural reconstruction at the sacral level. A comparison with a cohort of 81 patients subjected to conventional sutured dural closure revealed that 19 individuals (23.46%) developed cerebrospinal fluid fistulas. Treatment modalities varied, with six cases undergoing surgical intervention, six receiving external lumbar drainage, and one requiring both surgical intervention and external lumbar drainage, while spontaneous resolution was observed in six cases.

Data were described using a number and a percentage if categorical or a mean and a standard deviation if continuous. The prevalence of a fistula in the sample was described using a number, a percentage, and a 95% confidence interval. The association with the presence of a fistula were explored with logistic regression analysis, and the results were expressed using an OR with 95% confidence interval. A significance threshold was set at 0.05. All analyses were performed with Stata version 18 (StataCorp. 2023. Stata Statistical Software: Release 18. College Station, TX, USA: StataCorp LLC).

No statistically significant correlation was found between the number of fistulas and the parameters under examination. However, an increased correlation was observed between CSF fistula occurrence and large dural openings >3 vertebrae (OR = 2.00), the lumbosacral segment (OR = 2.31), and tumor histotypes such as schwannoma (OR = 2.00), filum terminale ependymoma (OR = 2.27), and ependymoma (OR = 2.33) (Table 1).

## 4. Discussion

Closure of the spinal dura mater following intradural tumor excision surgery poses a challenge for the surgeon due to the risk of postoperative fistula [1]. This can lead to various complications including infectious complications ranging from wound infections to meningitis, intracranial hypotension and hemorrhage, nerve root compression syndromes, and back pain [8], resulting in increased hospitalization times and costs [9]. The incidence of a fistula following intradural spinal surgery ranges from 2% to 34% [10]. Various methods and procedures for dural closure have been developed [11,12,13]; these may include direct suture, autologous tissue (muscle or fat), or synthetic artificial materials [3,4,14]. Additionally, reinforcement with Tachoseal^®^ or fibrin glue can be applied [15,16].

Direct suture is the primary goal, which has a failure rate of approximately 17.5% [2]. However, as was highlighted in the systematic review by Choi et al., this failure rate is significantly reduced to a range of 5.5% to 13.7% through the adjunctive use of various materials, which decreases the incidence of CSF leak [2].

Over the years, a new dural closure method using non-penetrating metal clips [17], initially employed in vascular surgery was invented [7]. This method was first applied in pediatric and then adult spine surgeries [18,19].

Our experience involved 272 patients undergoing spinal surgery for intradural pathology where dural reconstruction was performed using the AnastoClip^®^ Closure System (AnastoClip^®^ Vessel Closure System, LeMaitre Vascular, Inc., Burlington, MA, USA), as is detailed in Appendix A.

**Figure 2:**
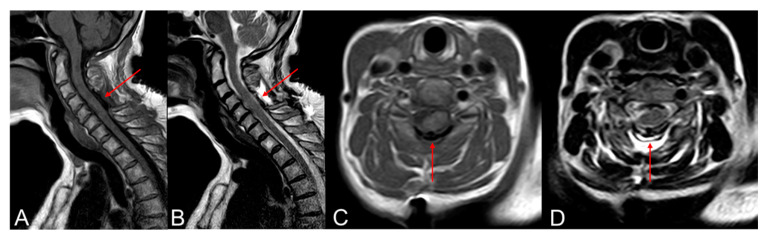
Titanium non-penetrating clips in the postoperative MRI (arrow). The use of clips does not compromise the quality of postoperative MRI images and does not show significant artifacts. T1 sagittal and axial plane (**A**,**C**). T2 sagittal and axial plane (**B**,**D**).

Numerous investigations have examined the efficacy of these clips. Non-penetrating titanium clips offer a dural closure that exhibits immediate hydrostatic strength like intact dura, whereas suturing with either material proved notably less resilient. Moreover, the application of titanium clips demonstrated a more rapid procedure compared to suture repair [20]. Kiyoshi I. et al. conducted experimental studies comparing the hydrostatic pressure tolerance between non-penetrating titanium clips and conventional sutures. They found that the leakage pressure in the non-penetrating titanium clip group was 1.8 times higher than that in the suture group. Furthermore, the clips did not create holes, and fluid leakage occurred between them, while in the suture group, leakage occurred at the suture holes. This led them to conclude that the interrupted placement of non-penetrating titanium clips allows for dural closure without creating holes, leading to improved initial leakage pressure and reduced postoperative CSF leakage following spinal surgery [21]. Furthermore, clips suture for dural repair in a relevant animal model displayed significantly less extensive acute and chronic inflammation, foreign body reactions, and meningoneural adhesions compared to suture [22]. Studies evaluating the metallic artifacts caused by the AnastoClips^®^ in postoperative neuroimaging have concluded that no significant alterations in evaluation quality occur postoperatively (Figure 2) [23].

Two significant series are found in the literature, one focused on pediatric cases and the other on adults. Shane S. et al. presented a case series of 152 pediatric patients undergoing lumbar durotomy, followed by dural closure using the AnastoClip^®^ non-penetrating titanium clip closure system. Postoperative CSF leakage occurred in 1.32% of patients at 11 and 18 days [18]. Timothy J. et al. reported on 58 patients, with a mean age of 53 years (range: 21–88), treated for spinal intradural tumors with dural closure using non-penetrating titanium clips. CSF leakage occurred in 13.7% of patients [19].

Our results confirm that the use of clips is both safe and effective in reducing the risk of CSF fistulas. Their application method is simple, operator-independent, and does not require a steep learning curve. Compared to traditional suturing, clip appliers require significantly less space for effective use, enabling a narrower surgical corridor. Clips can also be applied in closer proximity, minimizing the dead space typically created by suture threads. Additionally, the use of clips significantly reduces the time needed for dural closure, by approximately half in single-level dural openings and up to one-third in multi-level openings.

Our case series did not reveal a statistically significant correlation between postoperative fistula risk and factors such as high BMI, lumbar level, large dural openings, or specific histotypes. However, when compared to patients whose closures were performed with sutures, we observed a lower fistula rate: 11.76% with clips versus 23.46% with sutures. Notably, these results are consistent with findings reported in the adult population (11.76% vs. 13.70%).

Despite these promising outcomes, one limitation of our study is the high variability within the sample. Other limitations include the absence of randomization and the exclusion of patients who had previously undergone intradural surgery or those with dural substance loss requiring the application of dural patches.

## 5. Conclusions

The prevention of CSF fistulas following spinal surgery remains a significant challenge in contemporary practice. The use of non-penetrating titanium clips has been extensively documented in the literature since the early 2000s, with biomechanical studies highlighting their advantages and limitations. Based on our experience with the largest clinical series of adult patients, non-penetrating titanium clips are a safe and effective alternative to sutures, with a fistula rate of 11.76%, aligning with results reported in the literature. They reduce operative time, preserve dural integrity, and result in fewer CSF fistulas compared to traditional suturing techniques, without introducing artifacts in postoperative imaging. Their adoption could represent a significant advancement in the prevention of CSF fistulas in spinal surgery, resulting in improved patient recovery.

## Figures and Tables

**Table 1:** Statistical analysis of the correlation between CSF leak and patient data.

	All	Fistula	No Fistula	OR (95% CI)	*p*
**n**	272	32	240		
**Sex (M)**	120 (44.12%)	13 (40.62%)	107 (44.58%)	0.85 (0.40–1.80)	0.672
**BMI**	24.8 ± 4.4	24.4 ± 3.1	24.9 ± 4.5		
Low	13 (4.78%)	1 (3.12%)	12 (5.00%)	0.56 (0.07–4.61)	0.594
Normal	140 (51.47%)	18 (56.25%)	122 (50.83%)	1	
Over	89 (32.72%)	12 (37.50%)	77 (32.08%)	1.06 (0.48–2.31)	0.891
Obese	30 (11.03%)	1 (3.12%)	29 (12.08%)	0.23 (0.03–1.82)	0.165
**Age**	55.6 ± 15.6	53.5 ± 15.5	55.9 ± 15.7	0.99 (0.97–1.01)	0.408
**N involved vertebrae**					
1	69 (25.37%)	9 (28.12%)	60 (25.00%)	1	
2	160 (58.82%)	17 (53.12%)	143 (59.58%)	0.79 (0.33–1.88)	0.597
3	30 (11.03%)	3 (9.38%)	27 (11.25%)	0.74 (0.19–2.95)	0.671
>3	13 (4.95%)	3 (9.38%)	10 (4.17%)	2.00 (0.46–8.68)	0.355
**Spine level**					
Cervical	57 (20.96%)	5 (15.62%)	52 (21.67%)	1	
Thoracic	105 (38.60%)	7 (21.88%)	98 (40.83%)	0.74 (0.22–2.46)	0.626
Lumbar-sacral	110 (40.44%)	20 (62.50%)	90 (37.50%)	2.31 (0.82–6.52)	0.114
**Histology**					
Meningioma	75 (27.57%)	5 (15.62%)	70 (29.17%)	1	
Schwannoma	96 (35.29%)	12 (37.50%)	84 (35.00%)	2.00 (0.67–5.95)	0.213
Ependymoma	21 (7.72%)	3 (9.38%)	18 (7.50%)	2.33 (0.51–10.69)	0.275
Ependymoma filum	43 (15.81%)	6 (18.75%)	37 (15.42%)	2.27 (0.65–7.94)	0.199
Others	37 (13.60%)	6 (18.75%)	31 (12.92%)	2.71 (0.77–9.55)	0.121

## Data Availability

Data are included in this study.

## Appendix A

*Closure technique for intentional durotomy*. We use the Prolene 6-0 curved needle suture thread for suspension of dura mater during dural opening and to affix two to three stiches for the closure, as is described in previously published papers on the use of titanium clips (two at the extremities of dural opening and one or two eventual stiches in the middle depending on the dural opening size) [20,21]. This approach has the advantage that the needle has the same diameter as the thread. Subsequently, we apply titanium clips for dural closure using a specialized applicator, as is described in the technical note by P. Marks et al. and the experimental studies by K. Ito et al., followed by the application of a sealant to ensure a watertight closure [17,21]. At the end, we ask the anesthetist to perform a Valsalva maneuver to check eventual tears. Finally, we close the muscular plane with detached stiches and fascial plane with tight running suture or detached stiches. The subcutaneous and cutaneous plane is performed with suture and/or staples. Usually, we use subfascial hematic for draining expect in very small openings.

*Postoperative cares protocol*. The subfascial drainage is removed the morning after surgery with partial mobilization for physiological function; the second day after surgery, the full mobilization is permitted. A postoperative MRI is usually performed 24 to 48 h after surgery. The hospital discharge is usually 5 to 7 days after surgery without neurological deficits. In the case of neurological deficits, the patients can be granted some days of rehabilitation in the neurosurgical ward (less than 7 days) or they can be transferred to the rehabilitation ward in our hospital or in other medical centers. If during the hospital stay, the patient develops a CSF collection, we perform, in order, (1) aspiration and compressive medication, (2) the Trendelenburg position, and (3) eventually CSF diversion by external spinal drainage. Only if all these measures fail, do we perform revision surgery. Normally, cutaneous stiches were removed 10 to 12 days after surgery. In the case of CSF collection/fistula occurring after the hospital discharge, we perform the same steps in an outpatient’s regimen and in the case of persistency the patients are readmitted to the hospital and a new MRI scan is performed for decision making.
